# Supplementary material for: Young at risk-people in Maputo City, Mozambique, present a high willingness to participate in HIV trials: Results from an HIV vaccine preparedness cohort study
Source: PLoS One. 2021 Dec 2;16(12):e0260126. doi: 10.1371/journal.pone.0260126 (PMC8638929; doi:10.1371/journal.pone.0260126)
Supplement: S1 Table — (PDF) [file pone.0260126.s001.pdf]

RV-363 HIV/AIDS Awareness, Vaccine Knowledge and Willingness to Participate Questionnaire	
**Site** ☐ 1 ***CISPOC*** ☐ 2 ***CIDI***	**Visit:** ____________________<br>**Subject Number** ___ ___ ___ ___ ___ ___

INTERVIEWER TO READ THIS

Before we start, I would like to remind you about the interview process. Your participation in this interview and every aspect of the research study are completely voluntary. You may skip any question that you prefer not to answer, but I would appreciate if you answered all the questions. You can also ask me anytime to clarify questions that you do not understand, or you can decide to stop the interview at any time.

Any information you provide for this study will be kept confidential and cannot be shared with any individual, including your employer/boss, spouse, friends, or relatives. The responses that you provide to these questions will only be identified by a unique number, not by your name or any other identifying information.

Today I am going to ask you to tell me what you know about HIV infection, and some questions on what you know about vaccines. This questionnaire has only two sections.

If you have any questions please do not hesitate to ask me anytime as we go through the questions.

Thank you. Let us begin!

Time Interview Started: ___ ___:___ ___ *hh:mm (24 hour clock time)*

**Form Completed By:** ☐☐☐	**Date Completed** *DD/MON/YYYY* __ __/__ __ __/__ __ __ __

**** All Questions**	**OTHER (SPECIFY)**	**=**	**Responses which do not fit into any category.<br>Should occur in minimal cases.**
	**REFUSED TO ANSWER**	**=**	**Respondent refused to answer the question for any reason.<br>Should occur in minimal cases.**
	**DO NOT KNOW**	**=**	**Respondent states they do not know. He/She may actually know, but does not want to give the answer.<br>Should occur in minimal cases.**

Site: ☐ 1 ***CISPOC*** ☐ 2 ***CIDI*** **Subject Number** ___ ___ ___ ___ ___ ___ **Visit:** _________

ADMINISTRATIVE INFORMATION

**1. Visit Date**	☐☐ DAY	☐☐☐ MONTH	☐☐☐☐ YEAR

2. Volunteer's residence (Identify code and record code number in boxes)

District code : ☐☐☐ **Neighborhood Code:** ☐☐☐

INSTRUCTION FOR FORM COMPLETERS

**** **DO NOT** READ ANSWER CHOICES OUT ALOUD TO A PARTICIPANT UNLESS INSTRUCTED ****

WITH
***READ** SIGN*

Read

******HOWEVER **DO NOT** READ******

*'**REFUSED TO ANSWER**' and '**DO NOT KNOW**' answer choices*

***** FOR SOME QUESTIONS YOU WILL SEE THE **ARROW** SIGN FOLLOWED BY A LETTER **'Q'** AND **NUMBER** OR **SECTION.** IT IS INSTRUCTING YOU TO '**SKIP**' TO ANOTHER QUESTION OR SECTION. **PLEASE FOLLOW THE SKIP PATTERN CAREFULLY** ****

→Q *Means ***Skip*** to another **Question***

→SEC *Means ***Skip*** to the next **Section***

Site: ☐ 1 *CISPOC* ☐ 2 *CIDI* **Subject Number** ___ ___ ___ ___ ___ ___ **Visit:** _________

Section 1: AIDS AWARENESS

1.1	I am going to read you a list of some ways people believe you can become infected with HIV. Please tell me which of the ways you agree that you could become infected with the HIV virus.<br>**Read**<br>(CHECK ALL APPLICABLE ANSWERS)	☐ HAVING PENILE-VAGINAL SEX WITH SOMEONE WHO HAS THE HIV VIRUS<br>☐ PLACING YOUR MOUTH ON YOUR PARTNER'S GENITALS<br>☐ PLACING YOUR PENIS IN THE ANUS OR LETTING YOUR PARTNER PLACE HIS PENIS IN THE ANUS<br>☐ KISSING MOUTH TO MOUTH<br>☐ INJECTING DRUGS WITH SYRINGES OR NEEDLES, USED BY SOMEONE WHO HAS THE HIV VIRUS<br>☐ SHARING RAZORS, SCISSORS, NEEDLES WITH SOMEONE WHO HAS THE HIV VIRUS<br>☐ CONTACT WITH BODY EXCRETIONS FROM SOMEONE WITH THE HIV VIRUS<br>☐ GETTING BLOOD TRANSFUSION FROM SOMEONE WHO HAS THE HIV VIRUS<br>☐ A BABY BREAST FED BY MOTHER WHO HAS THE HIV VIRUS<br>☐ EXPERIMENTAL HIV VACCINES<br>☐ NONE OF THESE<br>☐ REFUSED TO ANSWER<br>☐ DO NOT KNOW
1.2	I am going to read a second list of some ways people believe you can become infected with HIV. Please tell me which of the ways you agree that you could become infected with the HIV virus.<br>**Read**<br>(CHECK ALL APPLICABLE ANSWERS)	☐ CONDOM<br>☐ WORKING NEAR SOMEONE WHO IS INFECTED WITH HIV<br>☐ SHARING EATING UTENSILS<br>☐ USING SAME TOILET WITH SOMEONE WHO HAS THE HIV VIRUS<br>☐ MOSQUITOES<br>☐ NONE OF THESE<br>☐ REFUSED TO ANSWER<br>☐ DO NOT KNOW

Site: ☐ 1 ***CISPOC*** ☐ 2 ***CIDI*** **Subject Number** ___ ___ ___ ___ ___ ___ **Visit:** _________

1.3	From this list that I am going to read, tell me which ways you think a person can avoid or prevent getting infected with HIV?<br><br>(CHECK ALL APPLICABLE ANSWERS)	**Read**<br>☐ HAVE SEX ONLY WITH HEALTHY-LOOKING PERSONS<br>☐ USE CONDOMS<br>☐ USE FAMILY PLANNING SPERMICIDES BEFORE SEX<br>☐ CLEANING/DOUCHING AFTER SEX<br>☐ WITHDRAW BEFORE EJACULATING<br>☐ ABSTAIN FROM SEX<br>☐ DON'T SHARE NEEDLES<br>☐ REFUSED TO ANSWER<br>☐ DO NOT KNOW
1.4	Can you tell by looking at someone if they have the HIV virus?<br>☐☐	NO 00<br>YES 01<br><br>REFUSED TO ANSWER 88<br>DO NOT KNOW 99
1.5	If you tested HIV positive and chose to disclose your status to others, which of the following things do you think would happen in your life?<br><br>(CHECK ALL APPLICABLE ANSWERS)	**Read**<br>☐ BREAK UP OF MARRIAGE<br>☐ BREAK UP OF SEXUAL RELATIONSHIPS<br>☐ PHYSICAL ABUSE BY SPOUSE/SEXUAL PARTNER<br>☐ INCREASED SUPPORT FROM SPOUSE/SEXUAL PARTNER<br>☐ DISCRIMINATION BY EMPLOYERS<br>☐ INCREASED SUPPORT FROM EMPLOYERS<br>☐ NEGLECTED BY FAMILY<br>☐ DISOWNED BY FAMILY<br>☐ INCREASED SUPPORT FROM FAMILY AND RELATIVES<br>☐ ESTRANGED FROM MY PEERS<br>☐ INCREASED SUPPORT FROM PEERS<br>☐ OTHER (SPECIFY)<br>______________________<br>☐ REFUSED TO ANSWER<br>☐ DO NOT KNOW

1.6	Prior to coming in for this study, which of the following ways did you receive information about HIV/AIDS?<br><br>(CHECK ALL APPLICABLE ANSWERS)	☐ NO INFORMATION ABOUT HIV/AIDS<br>☐ NEWSPAPER OR MAGAZINES<br>☐ RADIO<br>☐ TELEVISION<br>☐ FRIENDS/NEIGHBOURS<br>☐ FAMILY<br>☐ SCHOOL<br>☐ HEALTH PERSONNEL<br>☐ POSTER/PAMPHLET<br>☐ NGO OUTREACH<br>☐ INTERNET<br>☐ SITE RECRUITMENT STAFF<br>☐ COMMUNITY EVENT<br>☐ OTHER (SPECIFY)<br>________________________<br>☐ REFUSED TO ANSWER<br>☐ DO NOT KNOW	**Read**

Section 2: VACCINE KNOWLEDGE

I am now going to ask you some questions about vaccines. These questions are intended to assess your understanding of what vaccines are, and the purpose of vaccines. This information will help the study team design appropriate education materials on vaccines for people in your community.

2.1	Can you please tell me if the following statement is true or false?<br><br>"**A vaccine is meant to prevent illness**"<br><br>☐☐	FALSE 00<br>TRUE 01<br><br>REFUSED TO ANSWER 88<br>DO NOT KNOW 99	
2.2	Prior to coming to this study, have you ever received education/information on HIV vaccine research?<br><br>☐☐	NO 00<br>YES 01<br><br>REFUSED TO ANSWER 88<br>DO NOT KNOW 99	→**Sec.3**

2.3	From this list I'm going to read, which of these sources have you received information about HIV vaccine research?<br><br>(CHECK ALL APPLICABLE ANSWERS)	☐ HOSPITAL/CLINIC/HEALTH WORKER<br>☐ RADIO/TV<br>☐ NEWSPAPER/BROCHURE/MAGAZINES<br>☐ FRIEND/RELATIVE<br>☐ INTERNET<br>☐ NGO<br>☐ POSTER<br>☐ LEARNED FROM A VACCINE TRIALVOLUNTEER<br>☐ I WAS A VACCINE TRIAL VOLUNTEER<br>☐ RESEARCH CENTER<br>☐ SCHOOL<br>☐ OTHER (SPECIFY) ___________________________<br>☐ REFUSED TO ANSWER ☐ DO NOT KNOW<br>**Read**

Section 3: Willingness to Participate in Clinical Trials

Clinical trials are studies performed with human subjects to test new vaccines, new drugs or new devices. Clinical trials are used to determine whether these new biomedical interventions are safe and effective. For example, when we have a candidate HIV vaccine, we would test it experimentally in a controlled environment first. This process is called a clinical trial.

3.1	Would you be willing to participate in such a study to test an experimental HIV vaccine?<br>☐☐	NO	00	
		YES	01	→Q3.3
		SOMEONE ELSE WOULD DECIDE	02	
		REFUSED TO ANSWER	88	
		DO NOT KNOW	99	

3.2	Please tell us why you will not be willing to participate in a vaccine study?<br><br>(CHECK ALL APPLICABLE ANSWERS)	☐ FEAR OF NEEDLE<br>☐ FEAR OF GETTING HIV<br>☐ SPOUSE/SEXUAL PARTNER REFUSAL<br>☐ FEAR OF SIDE EFFECTS<br>☐ FEAR OF DEATH<br>☐ FEAR OF FETAL ABNORMALITIES<br>☐ TIME REQUIRED FOR VISITS<br>☐ FEAR OF TESTING HIV POSITIVE<br>☐ I KNOW I AM HIV POSITIVE<br>☐ FEAR OF DESCRIMINATION<br>☐ OTHER (SPECIFY)<br>___________________________<br>☐ REFUSED TO ANSWER<br>☐ DO NOT KNOW<br>**Read**

3.3	From this list I'm going to read, can you please choose some of personal advantages of participating in a study of an HIV vaccine?<br><br>**Read**<br><br>(CHECK ALL APPLICABLE ANSWERS)	☐ LEARN HOW TO AVOID RISKY BEHAVIOR<br>☐ FEEL PROTECTED FROM HIV INFECTION<br>☐ GET FREE HIV COUNSELING AND TESTING<br>☐ RECEIVE UPDATED INFORMATION ABOUT HIV/AIDS<br>☐ GET SMALL REIMBURSEMENT EVERY TIME I COME TO A STUDY VISIT<br>☐ BE TESTED FOR SEXUALLY TRANSMITTED INFECTION<br>☐ RECEIVE REGULAR HEALTH CARE RELATED TO RESEARCH<br>☐ GET FREE PREGNANCY TEST EVERY 3 MONTHS<br>☐ OTHER (SPECIFY)<br>_______________________________<br>☐ REFUSED TO ANSWER<br>☐ DO NOT KNOW

3.4 How likely or unlikely would you be to enroll in a research study of a new experimental HIV vaccine if:

Read

*(Use these responses to fill the question **below**)*

VERY UNLIKELY	00
SOMEWHAT UNLIKELY	01
SOMEWHAT LIKELY	02
VERY LIKELY	03
NOT APPLICABLE	77
REFUSED TO ANSWER	88
DO NOT KNOW	99

☐☐ a. YOU WERE REQUIRED TO COME TO THE CENTER ON A SPECIFIC DAY EVERY THREE MONTHS FOR TWO YEARS

☐☐ b. YOU WERE REQUIRED TO TALK TO A NURSE ABOUT YOUR EXPERIENCES IN THE STUDY

☐☐ c. YOU WERE REQUIRED TO BE TESTED FOR HIV EVERY THREE MONTHS

☐☐ d. YOU WERE REQUIRED TO BE INJECTED WITH A VACCINE CANDIDATE A FEW TIMES

☐☐ e. YOU WERE REQUIRED TO GIVE BLOOD SAMPLES EVERY TIME YOU CAME FOR A STUDY VISIT

☐☐ f. THE RESEARCH CENTER WERE OPEN ON THE WEEKEND (E.G. SATURDAYS)

☐☐ g. IF FEMALE: YOU WERE REQUIRED TO USE A CONTRACEPTIVE METHOD SUCH AS HORMONAL CONTRACEPTION WHILE YOU ARE IN THE STUDY

3.5 How important are the following factors in making a decision about participating in a research study of an experimental HIV vaccine?

Read

*(Use these responses to fill the question **below**)*

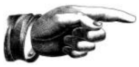

NOT IMPORTANT AT ALL	00
SOMEWHAT IMPORTANT	01
IMPORTANT	02
VERY IMPORTANT	03
REFUSED TO ANSWER	88
DO NOT KNOW	99

☐☐ a. I MAY BE HELPING FIND AN HIV VACCINE THAT WORKS

☐☐ b. I WOULD BE HELPING STOP THE HIV/AIDS EPIDEMIC

☐☐ c. MY FRIENDS AND FAMILY WOULD SUPPORT ME

☐☐ d. I WOULD MEET NEW PEOPLE

☐☐ e. I WOULD BE HELPING MY COMMUNITY

☐☐ f. I WOULD BE HELPING MY COUNTRY

☐☐ g. I WOULD BE HELPING ADVANCE HIV PREVENTION RESEARCH

☐☐ h. PEOPLE MAY THINK I HAVE HIV/AIDS

☐☐ i. PEOPLE MAY THINK THAT I AM AT RISK FOR HIV/AIDS

☐☐ j. PEOPLE MAY NOT WANT TO HAVE SEX WITH ME

☐☐ k. PEOPLE MAY REFUSE CONTACT WITH ME

3.6	Do you think a preventive HIV vaccine would be useful in controlling HIV infection? ☐☐	NO 00<br>YES 01<br><br>REFUSED TO ANSWER 88<br>DO NOT KNOW 99	

Ask the question below only at 'Exit Visit'

3.7	Can we contact you for future HIV vaccine studies? ☐☐	NO 00<br>YES 01	

CLOSING REMARKS

**** BEFORE YOU RELEASE THE VOLUNTEER, CHECK THAT ALL QUESTIONS ARE ANSWERED.**

Remember to record 'Time Interview Ended' at the bottom this page

INTERVIEWER TO READ THIS

Now I will be happy to let you know that I have finished all the questions. I would like to thank you for your patience, co-operation and honesty in answering the questions. I know it was a long and exhausting process for you, with some very sensitive and personal questions. We are grateful for your co-operation.

Again, I would like to remind you that all information you provided for this study will be kept confidential and will not be shared with any individual, including your employer/boss, spouse, friends or relatives. The responses that you provide to these questions will only be identified by a unique number, not by your name or any other identifying information.

THANK YOU VERY MUCH!

Time Interview Ended: ___ ___:___ ___ *hh:mm (24 hour clock time)*

1st Data Entry Initials/Date______________________ 2nd Data Entry Initials/Date_______________________
