# Supplementary material for: Young at risk-people in Maputo City, Mozambique, present a high willingness to participate in HIV trials: Results from an HIV vaccine preparedness cohort study
Source: PLoS One. 2021 Dec 2;16(12):e0260126. doi: 10.1371/journal.pone.0260126 (PMC8638929; doi:10.1371/journal.pone.0260126)
Supplement: Attachment — Submitted filename: WTP_RV363_PlosOne-Response to Reviewers.25.07.21.docx [file pone.0260126.s004.docx]

**Young at risk-people in Maputo City, Mozambique, present a high willingness to participate in HIV trials: results from an HIV vaccine preparedness cohort study**

I again congratulate the authors for such an interesting paper that helps to advance evidence of HIV vaccines. I also thank the authors for addressing the majority of issues in the manuscript. However, there are still some issues that need authors consideration as they are relevant. I leave to the Editor to decide the paper's outcome, to accept as it is, or to request another revision based on the reviewer's findings.

***R: We would like to take this opportunity to thank you for your comments and suggestions, which we believe will improve the manuscript. Please find the answers below.***

1. One issue is related to the interpretation of a borderline/ non-significant result on table 4. Significance and non-significance are based on an arbitrary cut-off of 5% and as such one needs to interpret according to the study context. Although I understand authors need report their bordeline result, I still believe authors may not report it as it is a significant finding, without a further explanation. Instead, they may need to discuss the meaning of this finding in terms of their impact or so. This may be an issue with sample size, but the meaning needs to be discussed. On page 19 (see below the text extract), the authors report the same findings as purely significant. This in my view, needs attention.

"*Among the 430 participants who stayed through the course of the study, results from the GEE* *265 binary logistic regression (screening visit and exit visit) showed that perceived risk for HIV* *266 infection (aOR 1.90, 95% CI: 0.99-3.63),… were associated with willingness to participate in HIV vaccine studies (Table 4)."*

*“k.Table 4: perceived risk for HIV infection was not significant. Authors may reconsider from including it as a factor associated with WTP in a vaccine trial R: We thank you for your comments. The Perceived risk was borderline significant p=0.053. We believe this trend should be considered.”*

**R: Thank you, this comment was well taken. We have clarified the marginally significant results by revising the text as follows:**

***“Among the 430 participants who stayed through the course of the study, results from the GEE binary logistic regression (screening visit and exit visit) showed that wanting to learn how to avoid risk behaviors (aOR 3.33, 95% CI: 1.61-6.86) and feeling protected against HIV infection (aOR 2.24, 95% CI: 1.07-4.7) were associated with willingness to participate in HIV vaccine studies. The perceived risk for HIV infection (aOR 1.90, 95% CI: 0.99-3.63) shows positive association, although remaining borderline significant (Table 4).”***

2. The second issue is related to the limitations of the study that needs to be clearly addressed. In my previous revision, I raised the issue of the generalizability of the study that needed to be discussed. However, the authors mentioned that this issue would be addressed in another paper, not the current one (see below the authors’ answer). I still believe that the issue of generalizability needs to be mentioned in this paper. There are other issues that were postponed to a future paper, but are also important in the current paper.

*“o.Lines 322-324: The authors discussed the limitations of the study. Can the authors discuss the generalizability of this study? What % of participants came from different suburban areas other than Polana Caniço? This is a relevant issue in this study as if I understood the majority o participants are from Polana Caniço area. R: We thank you for the comments. Data will be used in another manuscript (incidence paper).”*

**R: Our sincere apologies for not fully addressing the generalizability comment in the previous revision. We have now addressed the comment as suggested by including a paragraph on the generalizability of the study under a new ‘Limitations’ section of the manuscript.**

3. The third issue is acknowledging the self-presentation bias that another reviewer (#3) had raised but was not addressed by the authors. I also state that these type of studies are highly influenced by social desirability bias—this need to be considered in the limitations and actions to minimise it stated.

**R: We appreciate your comment, and our apologies for the oversight. The issue pertaining to self-presentation bias as well as social desirability bias have been addressed under the new ‘Limitations’ section of the manuscript.**
